# Health and adverse childhood experiences among homeless youth

**DOI:** 10.1186/s12887-021-02620-4

**Published:** 2021-04-07

**Authors:** Andrew J. Barnes, Amy L. Gower, Mollika Sajady, Katherine A. Lingras

**Affiliations:** 1 Developmental-Behavioral Pediatrics, Division of Clinical Behavioral Neuroscience, Department of Pediatrics, University of Minnesota, Minneapolis, MN USA; 2 Department of Pediatrics, University of Minnesota Medical School, 717 Delaware Street SE, Minneapolis, MN 55414 USA; 3 Children’s Minnesota Developmental Pediatrics, 2530 Chicago Ave S STE G055, Minneapolis, MN 55404 USA; 4 Department of Psychiatry and Behavioral Sciences, University of Minnesota, Minneapolis, MN USA

**Keywords:** Developmental origins of health and disease, Adverse childhood experiences, Homelessness

## Abstract

**Background:**

Homelessness is associated with health problems and with adverse childhood experiences (ACEs). The risk of chronic health conditions for homeless compared to housed youth, and how this risk interacts with ACEs remains unclear. This study investigated the relationship between ACEs, housing, and child health, and whether: 1) ACEs and health vary by housing context; 2) ACEs and homelessness confer independent health risks; and 3) ACEs interact with housing with regard to adolescent health.

**Methods:**

Using data from 119,254 8th–11th graders, we tested independent and joint effects of ACEs and past-year housing status (housed, family homelessness, unaccompanied homelessness) on overall health and chronic health conditions, controlling for sociodemographic covariates.

**Results:**

The prevalence of ACEs varied by housing status, with 34.1% of housed youth experiencing ≥1 ACE vs. 56.3% of family-homeless and 85.5% of unaccompanied-homeless youth. Health status varied similarly. Homelessness and ACEs were independently associated with low overall health and chronic health conditions, after adjusting for covariates. Compared to housed youth, both family-homeless youth and unaccompanied-homeless youth had increased odds of low overall health and chronic physical and/or mental health conditions. All ACE x housing-status interactions were significant (all *p* < 0.001), such that ACE-related health risks were moderated by housing status.

**Conclusions:**

ACEs and housing status independently predict health status during adolescence beyond other sociodemographic risks. Experiencing homelessness, whether unaccomapnied or with family, is associated with increased health risk, and every additional ACE increases this risk. Clinicians and health systems should advocate for policies that include stable housing as a protective factor.

## Background

Adverse Childhood Experiences (ACEs) are common during childhood, with over 32 million youth (45%) under 17 years having experienced at least one ACE in the United States [1]. Almost all youth with one or more ACEs report experiencing these stressful events before the age of 14 years [2]. Youth who experience homelessness have particularly high rates of ACEs and related cumulative psychosocial risk [3, 4]. However, research that compares the prevalence of ACEs between homeless and housed populations of youth is lacking.

The health consequences of ACEs are well-established in adults [5–9], and increasing evidence suggests that these pernicious effects often emerge during childhood and adolescence. Chronic health conditions that emerge during childhood in the context of ACEs occur along a risk gradient, with each additional ACE conferring additional risk [10–12]. For example, adolescents with ACEs are most likely to report poor overall health and are up to 1.5 times more likely to have obesity compared to peers without ACEs [13, 14]. Another recent study of adolescents involved in the juvenile justice system found that youth with four or more ACEs experienced significant mental health symptoms; these symptoms were buffered by high levels of internal resilience, school connectedness, family communication, and peer role models [15].

The known associations between youth homelessness and stressors such as ACEs are especially notable given the high prevalence of youth homelessness; on a single night in 2018, at least 180,000 families with children and 36,000 unaccompanied youth in the United States experienced homelessness [16]. Likewise, public schools identified over 1.3 million homeless youth in 2016 (91% of whom were homeless with family) – an increase of 4% over 2 years [17]. Youth experiencing family homelessness report more mental health problems than housed youth [18], including suicide attempts [19], even when controlling for family income. In addition, youth experiencing unaccompanied homelessness are at high risk of victimization, substance abuse, infectious diseases, and mental health problems [20]. While factors such as positive parenting relationships and increased parent responsiveness can moderate trauma symptoms and emotional-behavioral concerns in children experiencing homelessness [19, 21], it remains unclear how housing contexts might be associated with health among youth at high psychosocial risk due to ACEs.

There are no studies that we are aware of that have compared the risk of chronic health conditions among homeless and housed youth in the context of ACEs. We aimed to investigate how ACEs and housing relate to child health by determining whether ACEs and child health vary by housing context; if ACEs and homelessness confer independent risks to health; and how ACEs interact with housing contexts vis a vis adolescent health. We hypothesized that youth experiencing unaccompanied homelessness would have more chronic health conditions and ACEs compared to those who are homeless with family; ACEs and homelessness would be independently associated with chronic health conditions in youth; and that health problems would increase with each additional ACE but that this risk gradient would be moderated by consistent housing.

## Methods

### Study design and subjects

Data come from the 2016 Minnesota Student Survey (MSS), a cross-sectional state-wide surveillance survey of youth health, risk, and protective factors offered triennially to every public and charter school serving 5th, 8th, 9th, and 11th grades in Minnesota. In keeping with state law, passive parental consent is used (< 1% opt-out rate) and schools administer the survey via paper and pencil survey or online, at their discretion (the proportion of participants using each mode is not available). Approximately 2% of completed surveys are discarded due to highly implausible or incomplete data. In 2016, 85% of school districts contributed data from at least one grade. Because some items necessary for the current analysis were not asked of 5th graders, the current study uses data from 119,254 students in 8th, 9th, and 11th grade. The University of Minnesota Institutional Review Board determined this secondary analysis of anonymous data did not meet the definition of human subject research and was therefore exempt from further ethical review and approval.

### Measures

#### Adverse childhood experiences

A total of eight yes/no items were used to assess the presence of six ACEs: psychological abuse (1 item), physical abuse (1 item), sexual abuse (2 items), familial substance abuse (2 items), domestic violence (1 item), and parental incarceration (1 item). Full details are described elsewhere [13]. In brief, for each ACE with two items, endorsement of either item was considered to indicate the presence of that ACE [22]. A count of the number of ACEs was calculated, creating an index representing 0 to 6 possible ACEs.

#### Homelessness

One item assessed with three response options assessed housing status: “During the past 12 months, have you stayed in a shelter, somewhere not intended as a place to live, or someone else’s home because you had no other place to stay?” Response options included no; yes, with my parents or an adult family member; and yes, on my own without any adult family members. Responses were recoded to indicate 1) no past year homelessness; 2) past year homelessness with family only; 3) past year homeless alone, regardless of homeless with family response. Both for parsimonious interpretability of results and because experiencing both family- and non-family-homelessness in the past year likely represents a level of housing instability that supercedes family homelessness, we decided, a priori, to include the 228 students who experienced both forms of homelessness in the homeless-alone category.

#### Health

Students self-reported their overall health as excellent, very good, good, fair, or poor, which was dichotomized into excellent/very good compared to good/fair/poor [23, 24]. Two questions assessed chronic health problems. Students reported the presence or absence of 1) “physical disabilities or long term-health problems (such as asthma, cancer, diabetes, epilepsy, or something else)?” or 2) “long-term mental health, behavioral, or emotional problems?” For both questions, long-term was defined as “lasting 6 months or more.” Responses were combined to create four mutually exclusive groups: no chronic health problems, only chronic physical health problems, only chronic mental health problems, and both types of chronic health problems.

##### Sociodemographic covariates

Additionally, we measured multiple sociodemographic variables with well-established associations with ACEs, housing status, and/or health [25–27]. Students reported their sex (male/female) and grade (8th, 9th, or 11th). Two questions were combined to create race/ethnicity groups: Hispanic and Non-Hispanic White; Black/African/African American; Asian/Pacific Islander; American Indian; and multiracial. We included receipt of free or reduced-price lunch at school as a binary marker for low-income status. Finally, we created a binary variable for location (Minneapolis-St. Paul metropolitan area versus all other non-metropolitan areas of Minnesota).

### Statistical analysis

Descriptive statistics were computed to describe the sample, stratified by housing status to show the distribution of sociodemographic subgroups and ACEs across the three categories of housing. Chi-square tests evaluated bivariate associations between housing status and both overall health and chronic health problems, after ensuring assumptions for Chi-square tests were met (i.e., mutually exclusive categories, independent samples, no cell with an expected value of < 1, and expected value of ≥5 for at least 80% of cells), to determine variable selection for two multivariable analyses. First, we evaluated the simultaneous, independent effects of housing status and ACEs on health using logistic regression. Housing status was dummy coded to examine the effects of homelessness with family and homeless alone, compared to the reference group of youth with consistent housing. Second, the interaction between ACEs and housing status was added to the regression to assess moderation. Because all interactions were significant, the sample was stratified by housing status, and multifactor ANOVAs were conducted to probe moderation effects [28]. Although ANOVA and other linear models were designed for normally distributed variables, they have been shown to be valid approaches for analysis of large datasets (*n* > 1000), regardless of the distribution. This approach allowed us to compare all three housing status groups to one another, rather than relying on comparisons to a control group. Adjusted predicted means are interpreted as predicted prevalence estimates for each group. All multivariable regressions controlled for sex, grade, race/ethnicity, free/reduced price lunch, and metropolitan location. Given the very large sample size, missing data were excluded listwise. Alpha was set to 0.05 for all analyses, and IBM SPSS v25 was used for data analysis.

## Results

### Prevalence of ACEs and health problems

The sample is described in Table 1; missing data ranged from 0% (biological sex) to 6.0% (ACEs). Participants were roughly evenly split by sex and grade, and race/ethnicity distributions were consistent with the demographics of Minnesota youth. The distribution of ACEs varied by housing status, with a flatter and higher-prevalence distribution for youth who were homeless alone than for youth who were homeless with family, and a less-prevalent skew among those with consistent housing. The median number of ACEs was 0 for housed youth (interquartile range (IQR) = 1); 1 for youth were homeless with family (IQR = 2); and 2 for youth who were homeless alone (IQR = 3). The prevalence of health problems also varied by housing status (Table 2). For all but chronic physical health problems, consistently-housed youth reported the best health in unadjusted analyses; those who were homeless with family reported intermediate levels of health problems; and students who were homeless alone had the highest frequency of health problems.

**Table 1:** Sample Characteristics by Housing Status

	Housed *n* = 112,750 (94.5%)	Family Homeless *n* = 5273 (4.4%)	Unaccompanied Homeless *n* = 1231 (1.0%)
Sex			
Male	55,466 (49.3%)	2883 (54.9%)	705 (57.7%)
Female	57,043 (50.7%)	2364 (45.1%)	516 (42.3%)
Grade			
8th	39,643 (35.2%)	2530 (48.0%)	371 (30.1%)
9th	40,191 (35.6%)	1876 (35.6%)	391 (31.8%)
11th	32,916 (29.2%)	867 (16.4%)	469 (38.1%)
Free/reduced lunch	28,970 (25.9%)	2605 (49.9%)	583 (47.7%)
Metro	59,636 (52.9%)	2726 (51.7%)	599 (48.7%)
Race/ethnicity			
NH American Indian	1210 (1.1%)	137 (2.6%)	51 (4.2%)
NH Asian/Pacific Isl.	6409 (5.7%)	601 (11.5%)	57 (4.7%)
NH Black	5988 (5.3%)	460 (8.8%)	93 (7.7%)
NH Multiracial	8090 (7.2%)	485 (9.3%)	170 (14.0%)
Hispanic	10,013 (8.9%)	779 (14.9%)	164 (13.5%)
NH White	80,306 (71.7%)	2765 (52.9%)	679 (55.9%)
ACEs			
0 ACEs	74,002 (65.9%)	2287 (43.7%)	177 (14.5%)
1 ACE	21,315 (19.0%)	1173 (22.4%)	244 (20.0%)
2 ACEs	9408 (8.4%)	719 (13.7%)	193 (15.8%)
3 ACEs	4545 (4.0%)	509 (9.7%)	205 (16.8%)
4 ACEs	2057 (1.8%)	303 (5.8%)	178 (14.6%)
5 ACEs	894 (0.8%)	172 (3.3%)	161 (13.2%)
6 ACEs	152 (0.1%)	67 (1.3%)	62 (5.1%)

*ACE* Adverse Childhood Experience, *NH* Non-Hispanic

**Table 2:** Chi-Square Test of Associations between Overall Health and Chronic Health Problems by Housing Status

	Housed	Family Homeless	Unaccompanied Homeless
Chronic Health Status*			
No Chronic Problems	81,060 (72.6%)	3341 (64.2%)	490 (40.4%)
Chronic Mental Health	13,592 (12.2%)	871 (16.7%)	352 (29.0%)
Chronic Physical Health	11,648 (10.4%)	563 (10.8%)	118 (9.7%)
Both Chronic Problems	5384 (4.8%)	433 (8.3%)	253 (20.9%)
Overall Health*			
Good/Fair/Poor	35,649 (31.7%)	2239 (42.7%)	686 (56.0%)
Excellent/Very Good	76,817 (68.3%)	3009 (57.3%)	539 (44.0%)

*Chi square test of association indicated significant differences between groups, *p* < 0.05

### Associations between ACEs, homelessness, and health

Housing status and ACEs independently contributed to overall health and chronic health problems in multivariable logistic regressions examining independent effects of housing status and ACEs on health, controlling for sex, grade, race/ethnicity, free/reduced price lunch, and school metropolitan location (Table 3). Students who were homeless alone had higher adjusted odds of chronic mental health problems, and co-occurring chronic mental and physical health problems, than youth with consistent housing.

**Table 3:** Logistic Regressions Predicting Health Problems based on Homeless Status and ACEs

	Good/Fair/Poor Overall Health	Chronic Physical Health vs. No Chronic Problems	Chronic Mental Health vs. No Chronic Problems	Chronic Both vs. No Chronic Problems
Housed	1.00	1.00	1.00	1.00
Family Homeless	1.19 (1.12, 1.27)	1.11 (1.02, 1.23)	1.28 (1.17, 1.39)	1.44 (1.28, 1.62)
Unaccompanied Homeless	1.34 (1.18, 1.51)	1.35 (1.09, 1.67)	2.03 (1.73, 2.38)	2.48 (2.48, 3.58)
ACEs count	1.40 (1.38, 1.41)	1.16 (1.14, 1.18)	1.65 (1.63, 1.68)	1.74 (1.70, 1.78)

Analyses controlled for sex, grade, race/ethnicity, free/reduced price lunch qualification, and metropolitan location. Results presented are adjusted odds ratios with 95% confidence intervals; all were significant at *p* < 0.05

### Moderation of health Risk of ACEs by housing status

**Fig. 1:**
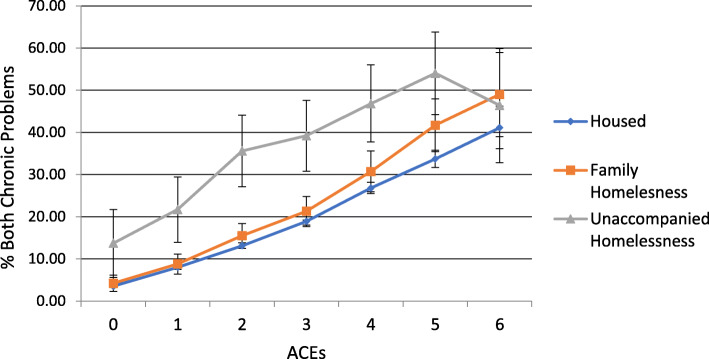
Prevalence of Chronic Physical and Mental Health Problems by ACEs and Housing Status

**Fig. 2:**
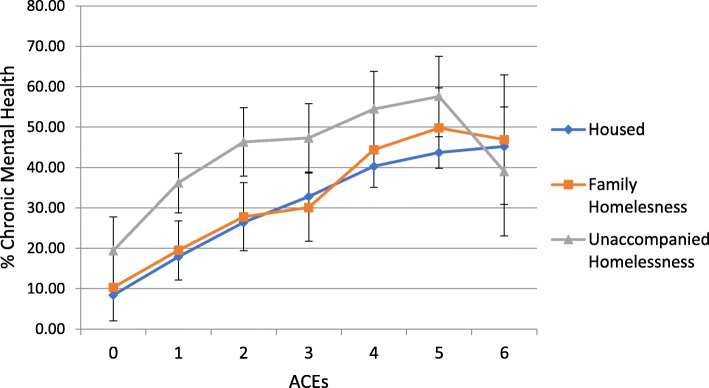
Prevalence of Chronic Mental Health Problems by ACEs and Housing Status

**Fig. 3:**
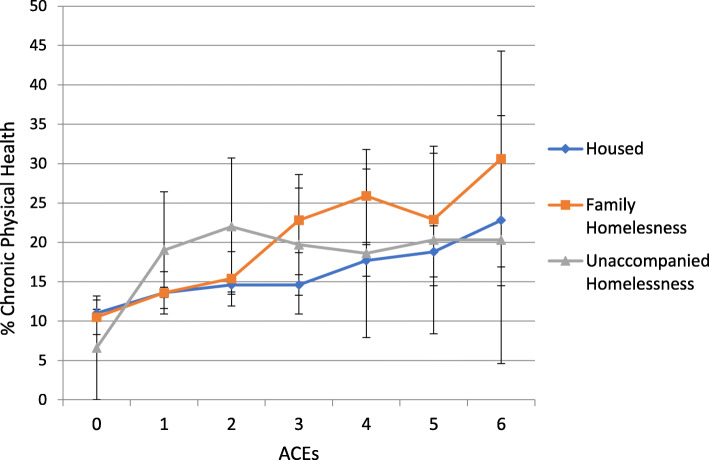
Prevalence of Chronic Physical Health Problems by ACEs and Housing Status

**Fig. 4:**
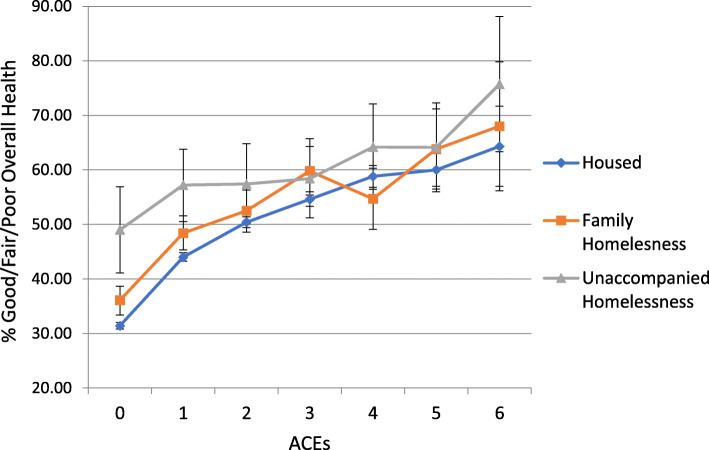
Prevalence of Good/Fair/Poor Health by ACEs and Housing Status

Health risks increased by ACEs regardless of housing status (Figs. 1, 2, 3 and 4). Because statistical interactions between ACEs and housing were significant for all health outcomes, we probed how housing status moderated the associations between ACEs and health. For youth with 0–5 ACEs, consistent housing and family homelessness were associated with lower prevalence of co-occurring chronic physical and mental health problems (Fig. 1) and lower prevalence of mental health problems (Fig. 2) compared to their prevalence among youth who were homeless alone. For youth with 0–2 or > 4 ACEs, physical health did not differ by housing status, but among those with 3–4 ACEs, consistent housing was associated with lower prevalence of physical health problems than family homelessness (Fig. 3). For youth with < 2 ACEs, consistent housing was associated with the highest levels of overall health (Fig. 4).

## Discussion

This study documents differential health risks among youth who were homeless with or without a family member (i.e., unaccompanied) during the past year in the context of ACEs. Youth experiencing homelessness were more likely to have health problems, ranging from 10% increased odds of physical health conditions among youth who were homeless with a family member, to over 2 times the odds of co-occurring chronic physical and mental conditions among unaccompanied-homeless youth. Cumulative psychosocial risk was much higher among these youth compared to those with consistent housing – a majority of youth who were homeless with a family member had 1 or more ACEs, and nearly 1 in 5 of youth who were homeless unaccompanied had over 4 ACEs. Each additional ACE, regardless of housing status, increased the odds of low overall health by 40%; chronic physical health conditions by 16%; chronic mental health conditions by 65%; and both chronic mental and physical health conditions by 74%. However, the health risks of homelessness remained elevated to a similar degree to the risks of ACEs even after controlling for ACEs along with sociodemographic factors that are associated with both homelessness and health. Furthermore, past-year homelessness compounded the health risks of ACEs to varying magnitudes depending upon past-year housing context (consistently-housed vs. homeless with or without family) and broad category of health outcome (overall health vs. chronic physical and/or mental health).

The finding that homelessness is associated with health problems is consistent with prior research [29, 30], and is the first to our knowledge to directly compare health between youth who were consistently housed and those who were homeless in the past year with family or unaccompanied. Similarly, the finding that health problems during adolescence are more prevalent with increasing ACEs aligns with research on pediatric obesity, behavioral health, and substance abuse [13, 31], as does the finding that health problems are associated with negative life events among young children experiencing concurrent family homelessness [4, 32, 33]. Our findings build upon this prior knowledge by establishing that ACEs and housing status pose independent and interacting problems for children’s health. Since health problems partially mediate long-term homelessness for adults who had ACEs [34, 35], our results imply that more intensive prevention, identification, and intensive treatment of chronic health problems in youth experiencing homelessness and ACEs could help “break the cycle” of homelessness across the lifespan.

Our results also highlight the complex interactions between housing, health, and ACEs. Few studies to date have examined risk gradients among subtypes of youth homelessness within the same sample. While we found that being homeless with family is associated with health risks, we also found that these youth may be somewhat better off from a health perspective than unaccompanied-homeless youth, and that youth with ACEs and unaccompanied homelessness have a very high prevalence of co-occurring health conditions. These results are consistent with a recent study of the intersection between youth who ran away from home and those who experienced homeless, showing that those who had both run away and been homeless were at greater odds of mental health problems than either group alone (those who ran away had intermediate odds) [36]. Overall, our findings affirm the protective nature of caregivers and families [37–41] and underscore the importance of ensuring that youth who have suffered adversity (e.g., maltreatment, neglect, or family dysfunction) are protected from becoming separated from supportive family members when faced with evictions or unsafe housing, humanitarian crises, disasters, war, or refugee/immigration policies that simultaneously threaten the integrity of families and the homes that allow health to thrive.

The strengths of this study include its use of a large, representative statewide sample, increasing statistical power to test hypothesized interactions between psychosocial factors among hard-to-reach populations of youth (i.e., those with high ACEs and/or homelessness). Additionally, the fact that these youth are enrolled and attending school provides one clear route for providing services and support. Limitations include the inability to draw causal inferences due to the cross-sectional sample; for example, health problems may increase the chances that youth experience homelessness. Furthermore, although MSS items assessing health and housing status have established associations with various outcomes in other studies [19, 42], these items are self-reported and have not yet been assessed for reliability and external validity. In particular, the validity of self-reported health status may be influenced by the number of chronic health conditions that are presented to respondents in health surveys [43]. Similarly, the housing items in the MSS do not assess other aspects of housing such as duration or frequency of homelessness, rent instability, or housing safety. Nevertheless, assessing homelessness using a broad definition, as done with the MSS, may be more accurate than head counts [44–46]. Similarly, the MSS sample selects for youth who attend school, so while it gives voice to many youth who are traditionally excluded from public health research (such as those experiencing homelessness), some of these youth who are most at risk (e.g., high ACEs; living on the street) might also be those most likely to be absent from school at the time of survey administration [47]. Given that our cohort was confined to one state (and reporting on only one year of housing status), longitudinal research of national samples is needed to better ascertain the directionality and generalizability of interactions between life experiences and health (e.g., before, during, and after episodes of homelessness). We are also limited to questions asked on the Minnesota Student Survey, limiting the covariates we could include in our models that have been associated with ACEs and/or mental health, such as juvenile criminal offending [48]. The administration mode (paper vs. online) of the MSS could also not be factored into our analyses. Finally, some of the stratified logistic regression models examining moderation of health by housing status had small sample sizes for youth with high ACEs, increasing the chances of type II errors (failing to reject false null hypotheses). We thus recommend that longitudinal nationally-representative surveys of youth health include items on both housing context and ACEs to inform program and policy development more precisely.

Our results have important clinical and policy implications for youth at high risk due to histories of maltreatment, neglect, and family dysfunction. Few pediatric clinicians ask about ACEs or document childhood adversity in the medical record [49]. Notably, ACEs surveys do not typically include housing status, so even if ACEs surveillance became a routine part of pediatric or community healthcare, housing experiences and/or needs might not be considered without additional screening. While some clinical systems have piloted methods to screen for social determinants of health, including housing and ACEs, efforts are widely variable and lack consistent protocols across the field [50–53]. As such efforts advance, our results – and those of others showing that even unstable or precarious housing is associated with health risks for children [54, 55] – suggest that the context of housing will be important to specify (i.e., within social determinants of health screening protocols that may include ACEs) because it is differentially associated with health risks.

Additionally, clinicians cite being hesitant to ask about some psychosocial topics due to lack of training in these areas as well as uncertainty as to how to resolve issues that arise from these questions [56, 57]. For example, within typical primary care settings, social work staff may be unavailable or overwhelmed, limited options may be available for on-site mental health care and related supports, and community-based options such as housing advocacy may not exist. Increasingly, efforts to link services such as behavioral health, food banks, housing services, and medical-legal partnerships within pediatric primary care attempt to bridge these gaps. Increased public support for such linkages and cross-sector collaborations such as school mental health and school-based clinics, along with public-private partnerships, will be essential in helping pediatric health care organizations integrate housing and ACEs screening, surveillance, and remediation into their processes that aim to address these and other social determinants of health.

In addition to trauma-informed care, advocacy is needed for “resilience-informed care” that incorporates protective factors both in clinical practice and social policies [41, 58]. Traditionally, policy work and medical care are focused on identifying and treating problems. Less attention is paid to what is going well, particularly in contexts of known risk, such as homelessness or ACEs. For instance, our results identify important nuances in health within the context of homelessness (i.e. level of ACEs and unaccompanied homeless versus homeless with family). These results illuminate that even within challenging situations, efforts to keep families together and ameliorate toxic stress can have important impacts on child health. Policies should aim to reflect this and recognize the protective impact of family relationships as well as other known facilitators of resilience.

## Conclusion

Youth experiencing ACEs or homelessness are at risk of poor overall health and chronic health conditions. Consistent housing in the past year has a generally protective effect on physical and mental health and can buffer the impact of ACEs during adolescence. Potential solutions include preventing ACEs through policy and public awareness; providing of trauma- and resilience-informed health and psychosocial services; and policies that support stable and sustainable family housing. Surveillance of ACEs at a community level (e.g., at the level of school districts, where data on homeless and highly mobile students is also kept) could be one approach to identifying which schools or student populations could benefit from targeted housing and health supports.

## Data Availability

The data that support the findings of this study are available from the Minnesota Department of Education, but restrictions apply to the availability of these data, which were used under license for the current study, and so are not publicly available. Data are however available from the authors upon reasonable request and with permission of the Minnesota Department of Education.

## Abbreviations

ACE: Adverse Childhood Experience
AOR: Adjusted Odds Ratio
CI: Confidence Interval
SD: Standard Deviation
MSS: Minnesota Student Survey
